# Comprehensive Analysis of Transcriptome and Metabolome Elucidates the Molecular Regulatory Mechanism of Salt Resistance in Roots of *Achnatherum inebrians* Mediated by *Epichloë gansuensis*

**DOI:** 10.3390/jof8101092

**Published:** 2022-10-17

**Authors:** Chao Wang, Rong Huang, Jianfeng Wang, Jie Jin, Kamran Malik, Xueli Niu, Rong Tang, Wenpeng Hou, Chen Cheng, Yinglong Liu, Jie Liu

**Affiliations:** 1 State Key Laboratory of Herbage Improvement and Grassland Agro-ecosystems, Center for Grassland Microbiome, Collaborative Innovation Center for Western Ecological Safety, Key Laboratory of Grassland Livestock Industry Innovation, Ministry of Agriculture and Rural Affairs, Engineering Ministry of Education, College of Pastoral Agriculture Science and Technology, Lanzhou University, Lanzhou 730000, China; 2 State Key Laboratory of Plateau Ecology and Agriculture, Qinghai Academy of Animal and Veterinary Sciences, Qinghai University, Xining 810016, China; 3 School of Life Science and Technology, Lingnan Normal University, Zhanjiang 524048, China

**Keywords:** *Epichloë gansuensis*, transcriptome, metabolome, differentially expressed genes, differentially expressed metabolite, salt tolerance

## Abstract

Salinization of soil is a major environmental risk factor to plant functions, leading to a reduction of productivity of crops and forage. *Epichloë gansuensis*, seed-borne endophytic fungi, establishes a mutualistic symbiotic relationship with *Achnatherum inebrians* and confers salt tolerance in the host plants. In this study, analysis of transcriptome and metabolome was used to explore the potential molecular mechanism underlying the salt-adaptation of *A. inebrians* roots mediated by *E. gansuensis*. We found that *E. gansuensis* played an important role in the gene expression of the host’s roots and regulated multiple pathways involved in amino acid metabolism, carbohydrate metabolism, TCA cycle, secondary metabolism, and lipid metabolism in the roots of *A. inebrians*. Importantly, *E. gansuensis* significantly induced the biological processes, including exocytosis, glycolytic process, fructose metabolic process, and potassium ion transport in roots of host plants at transcriptional levels, and altered the pathways, including inositol phosphate metabolism, galactose metabolism, starch, and sucrose metabolism at metabolite levels under NaCl stress. These findings provided insight into the molecular mechanism of salt resistance in roots of *A. inebrians* mediated by *E. gansuensis* and could drive progress in the cultivation of new salt-resistance breeds with endophytes.

## 1. Introduction

Soil degradation induced by salinity is putting global agriculture at risk, as it engulfs more than 6% of the world’s agricultural land, giving rise to a loss of USD 27.5 billion every year [1]. Moreover, this problem could become even worse due to backward irrigation practices, over-fertilization, and climate change [2]. Excessive salt is a detrimental factor that impedes plant growth and crop yield and directly threatens food security worldwide. Generally, salt stress is caused by high NaCl concentrations in soil, leading to ionic, osmotic, and secondary stresses in plants [3]. As sessile organisms, plants have evolved complex mechanisms for adaptation to highly saline conditions during their long-term evolution.

*Achnatherum inebrians*, a perennial grass, is mainly found in the natural alpine and subalpine grasslands of northwest China and plays a vital role in the ecological restoration of grasslands [4]. *Epichloë* endophytes are able to colonize the aerial tissues of host grasses, including seeds, stems, leaves, and peduncles, but with the exception of roots [5]. In addition, the presence of *Epichloë* endophytes confers increased resistance of host *A. inebrians* to salt stress [6,7], drought stress [8], low temperature [9], heavy metal stress [10], low nitrogen stress [11,12,13] and as well as improves the soil properties [14]. The increased resistance to various abiotic stress endowed by *Epichloë* endophytes has made *A. inebrians* thrive in these degraded grasslands of northwest China [15].

The maintenance of intracellular ion homeostasis is crucially important to the basic physiological events in living cells. Under salt stress, the high Na^+^ levels accumulated in the cytosol have negative effects on the metabolic enzymes, thus eventually resulting in the growth cessation or death of plants [16]. However, when exposed to high salinity conditions, the presence of *Epichloë* endophytes performs a positive effect on host plants via modulating ion redistribution, C:N:P stoichiometry, Ca^2+^ levels, photosynthetic parameters, chlorophyll content, enzyme activity of nitrogen metabolism, and nitrogen use efficiency, therefore contributing to the salt tolerance of host plants [14,17]. Additionally, based on metabolomic techniques, several crops colonizing with endophytes have been suggested to enhance systemic tolerance to salinity stress by inducing the levels of protective metabolites [18]. A variety of plant species accumulate nitrogen (N)-containing compounds, including amino acids and amides, that act as compatible solutes in response to osmotic stress as a result of high salinity levels; hence, nitrogen metabolism is centrally important for salt resistance [19]. A recent report suggests that N content, nitrogen utilization efficiency (NUE), the activities of glutamine synthetase (GS), nitrate reductase (NR), and nitrite reductase (NiR) were higher in *A. inebrians* plants with *E. gansuensis* (E+) than that of E- plants under NaCl stress [6]. In addition, the presence of *E. gansuensis* also increases plasma membrane H^+^-ATPase, glucose-6-phosphate dehydrogenase (G6PDH), superoxide dismutase (SOD) and catalase (CAT) activities to eliminate the detrimental effects of the reactive oxygen species (ROS) as well as Na^+^ ions in *A. inebrians* under NaCl stress [20].

As the symbionts, *Epichloë* endophytes uptake nutrition from the main source-host plants, and in turn, hosts acquire protection and competitiveness fed back by the fungi. Therefore, the beneficial interactions between host plants and *Epichloë* endophytes are indispensable. It has been extensively reported that the infection of *Epichloë* endophytes leads to the transcriptome reprogramming of host plants [9,21,22,23,24]. In our recent study, we evaluated the transcriptional response to NaCl stress in the leaves of *A. inebrians* based on Illumina sequencing and revealed that *E. gansuensis* up-regulated biology processes that were involved in the increased adaptation of host grasses to NaCl stress, such as cellular calcium ion homeostasis, proanthocyanidin biosynthesis, and brassinosteroid biosynthesis [7].

Although it has been well elucidated that *E. gansuensis* adjusts the trade-off between host plants’ growth and tolerance to NaCl stress via reprogramming the transcriptional events in leaves, the molecular mechanism for roots of *Epichloë*-infected *A. inebrians* in response to salt stress is still largely elusive. As the aerial *E. gansuensis* is absent in the roots, which are the primary tissues that exposed to highly saline conditions, it is fascinating to reveal the biological changes of roots mediated by above-ground *E. gansuensis* in response to NaCl stress. In this present study, the response in the roots of E+ and E− plants to 200 mM NaCl stress is assessed based on transcriptome analysis. Furthermore, a comparative metabolomic analysis was performed using the differentially expressed metabolites in roots of E+ and E− plants under 0 and 200 mM NaCl concentrations. The multi-omics analyses suggested that carbohydrate metabolism, TCA cycle, amino acid metabolism, and organic acid metabolism played important roles in the *E*. *gansuensis*-mediated resistance to NaCl stress in roots of *A. inebrians*.

## 2. Materials and Methods

### 2.1. Plant Growth and Salt Treatment

*Achnatherum inebrians* seedlings with or without *E. gansuensis* were prepared similarly to that described in our previous studies [6,7]. In brief, *E. gansuensis*-infected (E+) and *E. gansuensis*-free (E−) *A. inebrians* seedlings were sown in pots filled with sterile vermiculite (oven-dried at 150 °C for 3 h), and the vermiculite was saturated with ddH_2_O. After seed germination, each pot was irrigated with 150 mL 1/2 Hoagland solution and 200 mL ddH_2_O every week. After growth for 6 weeks, half of the E+ and E− seedlings were treated with 150 mL 1/2 Hoagland solution with 200 mM NaCl and 200 mL ddH_2_O; meanwhile, the other half of E+ and E− seedlings were irrigated with 150 mL 1/2 Hoagland solution and 200 mL ddH_2_O. Roots of *A. inebrians* from different treatments were simultaneously collected for the next experiments after 4-week treatment. All samples grew in a greenhouse with 26 ± 2 °C, 16 h light/8 h dark, and 42 ± 2% relative humidity.

### 2.2. RNA Extraction, PacBio Sequencing, Illumina Library Preparation, Transcriptome Sequencing and Gene Annotation

The total RNA of *A. inebrians* roots was isolated using a TianGen plant RNA easy fast kit (TIANGEN, Beijing, China; Cat no. DP452). The RNA samples were digested with RNase-free DNase I to remove the contaminating DNA. A bioanalyzer (Agilent Technologies, 2100, CA, USA) and a Nanodrop 2000 (ThermoFisher Scientific, MA, USA) were used to check the RNA integrity and quality, respectively. Subsequently, the full-length cDNAs were achieved by reverse transcription from high-quality RNA samples using a Takara SMARTer™ cDNA Synthesis Kit (ClonTech, CA, USA; Cat no. 634925), followed by size fractionation via the BluePippin Size Selection System (PacBio, Menlo Park, CA, USA). The size-selected cDNA (2 μg) was transformed into SMRTbell template using PacBio DNA Template Prep Kit 2.0 (PacBio, CA, USA). Then, the cDNA library was sequenced using the PacBio sequel platform.

For the raw data processing, the effective subreads were obtained by SMRTlink v8.0 with parameters of minlength = 50 and readScore = 0.65. Next, the cyclic consensus sequences (CCSs) were generated from the subread BAM files with parameters of min_length = 50, max_drop_fraction = 0.8, no_polish = TRUE, min_zscore = −9999.0, min_passes = 1, min_predicted_accuracy = 0.8, max_length = 15,000 [25]. CCS with poly(A) tail, 5’ and 3’ adaptors were defined as a full-length non-chimetric (FLNC) read. FLNC reads were processed by iterative clustering for error correction (ICE) algorithm to obtain the consensus sequences, followed by polishing with Quiver for subsequent analysis. Then, the data of Illumina sequencing were used to correct the polished consensus sequences via LoRDEC (v0.5.3). Finally, software CD-HIT was used to remove the redundant sequences based on 95% similarity.

Total RNA (2 μg) that was extracted from independent biological replicate was processed with NEBNext UltraTM RNA Library Prep Kit for Illumina (NEB, MA, USA) to generate the sequencing libraries. In order to collect the cDNA fragments with 240 bp in length preferentially, AMPure XP system (Beckman Coulter, CA, USA) was used to purify the library fragments. After being treated with USER Enzyme (NEB, MA, USA), size-selected, adaptor-ligated cDNA was used for PCR with Phusion High-Fidelity DNA polymerase. Finally, PCR products were purified, and the library quality was assessed by the Agilent Bioanalyzer 2100 system. The library was sequenced on an Illumina platform (Biomarker, Beijing, China). HISAT2 software (v 2.0.4) was used for reads alignment to the results of PacBio sequencing (major parameter: Hisat2-dta-p 6-max-intronlen 5,000,000), and the gene expression levels were estimated by Fragments Per Kilobase per Million (FPKM) via String Tie. Then, the results were further processed with BMKCloud online platform (www.biocloud.net (accessed on 12 February 2022)). Three biological replications for each treatment were designed in the RNA-seq experiment. The gene function annotation utilized the available databases, including NCBI’s non-redundant nucleotide and protein sequence databases, the protein families database (Pfam), Clusters of Orthologous Groups of proteins (KOG/COG), SWISS-PROT protein sequence database, Gene Ontology (GO) database, and KEGG Ortholog (KO) database.

### 2.3. Identification of Differentially Expressed Genes

A total of four comparative groups (0RE+ versus 0RE−, 200RE+ versus 200RE−, 200RE+ versus 0RE+, 200RE− versus 0RE−; 0RE+/− represents roots of *A. inebrians* with or without *E. gansuensis* at 0 mM NaCl, 200RE+/− represents roots of *A. inebrians* with or without *E. gansuensis* at 200 mM NaCl.) were established to identify the differentially expressed genes (DEGs). The detection of DEGs was performed by DEGSeq software v.1.5.6 with a rigorous algorithm [26]. The criteria for DEGs were a relative change threshold of 2-fold and false discovery rate (FDR) < 0.01.

### 2.4. GO Terms and KEGG Pathway Analysis

The Gene Ontology (GO) enrichment analysis based on the DEGs was carried out with the help of GOseq R packages, as described by Young et al. [27]. In order to conduct KEGG analysis for the DEGs, the freely available KOBAS system was used [28]. 

### 2.5. GC-MS Analysis

A previously described method by Hou et al. [13] was used to perform the GC-MS analysis for metabolites in *A. inebrians* roots. In brief, the 7890 gas chromatography (Agilent, CA, USA) coupled with a Pegasus HT^®^ time-of-flight mass spectrometer (LECO, MI, USA) was used for GC-MS analysis. The raw mass spectra data were processed via ChromaTOF software (versions 4.3×). The metabolites were identified based on the LECO-Fiehn Rtx5 database by matching the mass spectrum and retention index [29].

### 2.6. Statistical Analysis

SIMCA-P 14.0 software package was performed to plot the unsupervised principal component analysis (PCA) with 95% confidence ellipse and supervised orthogonal projections to latent structures discriminant analysis (OPLS-DA). To assess the model performances of OPLS-DA, R^2^X(cum) and Q^2^X(cum) values for the quantitative goodness-of-fit parameters and Q^2^(cum) values for the goodness-of-prediction parameters were calculated, respectively. Value of variable importance plot (VIP) indicated the weighted sum of the OPLS-DA’s squares. The significantly different metabolites were determined by criterion with *p* < 0.05 (Student’s *t*-test) and VIP > 1. Based on the significantly different metabolites, altered metabolic pathways were enriched via MetaboAnalyst (http://metaboanalyst.ca/ (accessed on 6 March 2022)).

## 3. Results

### 3.1. Salt Treatment Influences the Gene Transcript Levels in Roots of E. gansuensis-Infected and E. gansuensis-Free A. inebrians

Based on PacBio Sequel technology, we have acquired the full-length transcriptome data of *A. inebrians* [7]. For further transcriptome analysis, samples from four treatment groups (roots of *A. inebrians* with or without *E.gansuensis* at 0 mM NaCl, 0RE+/−; roots of *A. inebrians* with or without *E.gansuensis* at 200 mM NaCl, 200RE+/−) were sequenced by Illumina platform with three biological duplicates per group. In the Illumina result, a total of 77.38 GB of clean data was obtained after sequencing quality control, and the percentage of Q30 bases in each sample was not less than 94.24% (Supplementary Materials Table S1).

**Figure 1:**
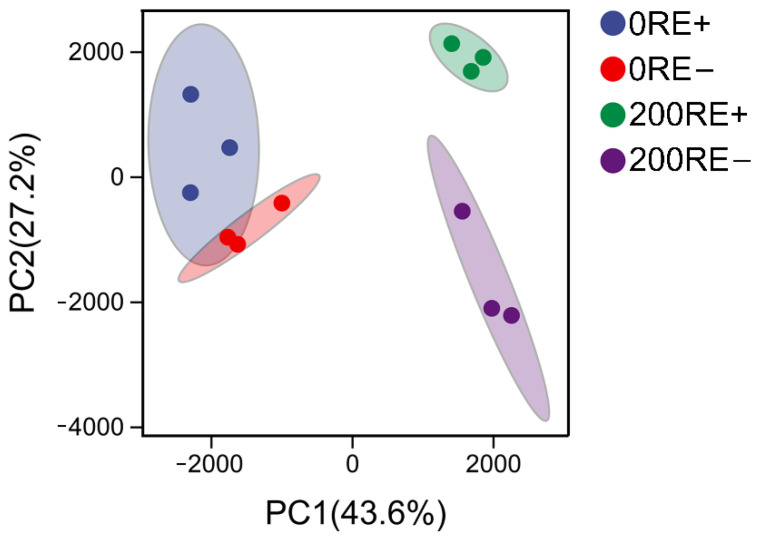
Principal component analysis (PCA) score plot showing the differences of transcriptome in roots between *E. gansuensis*-infected (E+) and *E. gansuensis*-free (E−) *A. inebrians* under 0 and 200 mM NaCl concentrations. 0RE+/−: roots of *A.inebrians* with or without *E. gansuensis* at 0 mM NaCl; 200RE+/−: roots of *A.inebrians* with or without *E. gansuensis* at 200 mM NaCl.

Roots of *A. inebrians* exhibited significant differences in transcriptional features after being treated with 200 mM NaCl. As shown in the score plot of principal component analysis (PCA), salinity significantly separated the transcriptional events in roots of E+ and E− *A. inebrians* along the PC1 axis (Figure 1). Additionally, distinct separation was found between 0RE+ and 0RE-. Interestingly, this difference rose to be significant when plants were exposed to NaCl stress, indicating *E. gansuensis* might play an important role in the transcriptional regulation of roots of *A. inebrians* under NaCl stress. Meanwhile, the correlation coefficient between samples of E+ and E− plants with NaCl treatment was lower than that under NaCl-free conditions (Supplementary Materials Figure S1), suggesting that NaCl increased the separation between E+ and Eࢤ plants at transcriptional levels.

### 3.2. Analysis of Differentially Expressed Genes (DEGs) in Roots of A. inebrians in Response to NaCl Stress

**Figure 2:**
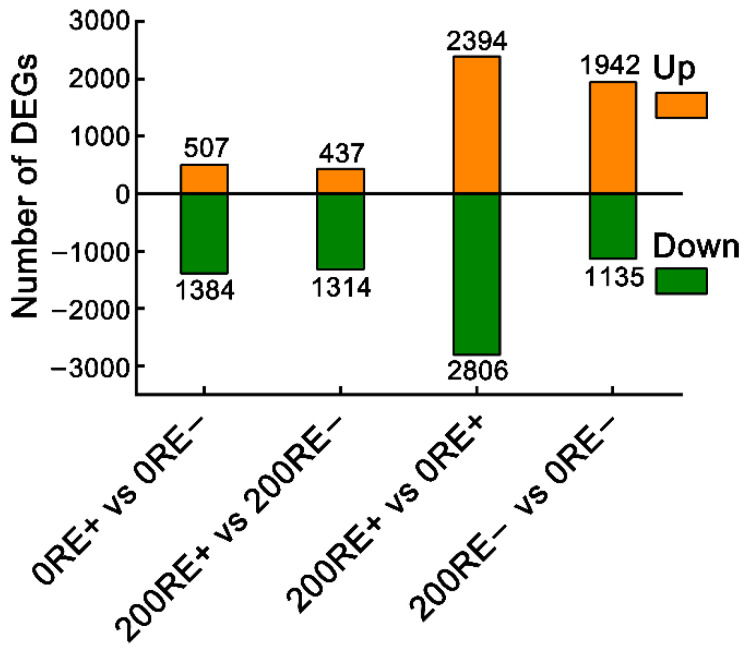
The number of up- and down-regulated DEGs in comparative groups of 0RE+ versus 0RE− (abbreviated as 0RE+ vs. 0RE−), 200RE+ vs. 200RE−, 200RE+ vs. 0RE+, and 200RE− vs. 0RE−.

Results of Illumina sequencing suggested that there were 1891 (507 up-regulated and 1384 down-regulated) DEGs, accounting for 6.8% of the total identified genes (27,850), between 0RE+ and 0RE− (Figure 2, Figures S2A and S3A); and 1751 (437 up-regulated and 1314 down-regulated) DEGs, accounting for 6.2% of the total identified genes (28,294), between 200RE+ and 200RE− (Figure 2, Figures S2B and S3B). Additionally, we also noted dramatic changes in the transcriptional response to NaCl stress, with 5200 DEGs (21.1% of the total identified genes (24,624); 2394 up-regulated and 2806 down-regulated) for E+ roots and 3077 DEGs (11.4% of the total identified genes (27,077); 1942 up-regulated and 1135 down-regulated) for roots of E− plants (Figure 2, Figures S2C,D and S3C,D). These results indicate that infection of *E. gansuensis* markedly reprograms the transcriptional events in the roots of host plants under salt stress.

### 3.3. E. gansuensis Regulates Multiple Biological Processes in the Roots of A. inebrians in Response to NaCl Stress

**Figure 3:**
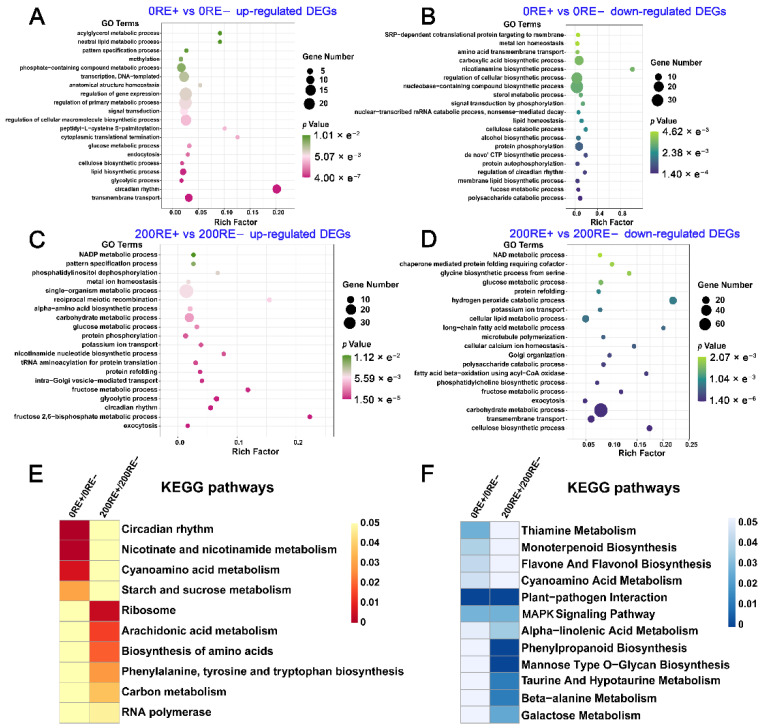
Gene ontology (GO) and KEGG pathway enrichment analysis of DEGs that identified between E+ and E− host roots at 0 and 200 mM NaCl concentrations. The GO terms of up-regulated (**A**) and down-regulated (**B**) DEGs between 0RE+ and 0RE−, The GO terms of up-regulated (**C**) and down-regulated (**D**) DEGs between 200RE+ and 200RE−. “Rich factor” represents the ratio of the enriched DEGs to the total genes annotated in the corresponding pathway. KEGG pathway enrichment analysis of up-regulated (**E**) and down-regulated (**F**) DEGs identified between roots of E+ and E− plants under 0 and 200 mM NaCl concentrations. Color panels show the *p*-value of KEGG pathway enrichment among the comparisons.

To further describe the biological roles of DEGs, we firstly carried out the gene ontology (GO) term enrichment analysis. As shown in Figure 3A, the categories of “transmembrane transport”, “circadian rhythm”, “glycolytic process”, “lipid biosynthetic process”, and “cellulose biosynthetic process” were significantly enriched among the up-regulated DEGs by *E. gansuensis* under 0 mM NaCl condition. In contrast, the down-regulated DEGs mediated by *E. gansuensis* under 0 mM NaCl levels were mainly concentrated in the biological processes, including “polysaccharide catabolic process”, “fucose metabolic process”, and “protein autophosphorylation” (Figure 3B). Furthermore, the GO enrichment analysis of DEGs suggested that *E. gansuensis* significantly induced “fructose 2,6-bisphosphate metabolic process”, “glycolytic process”, “circadian rhythm”, and “protein phosphorylation” under 200 mM NaCl stress (Figure 3C). Interestingly, biological processes involved in the “exocytosis”, “potassium ion transport”, “carbohydrate metabolic process”, and “fructose metabolic process” were commonly enriched in the up- and down-regulated DEGs between 200RE+ and 200RE- (Figure 3C,D). These results suggest that, when exposed to salt stress, *E. gansuensis* might improve the tolerance of roots of *A. inebrians* via regulating carbohydrate metabolism, activating signal transduction, and balancing potassium homeostasis.

The results of KEGG pathway enrichment analysis showed that categories of “circadian rhythm”, “nicotinate and nicotinamide metabolism”, “cyanoamino acid metabolism” and “starch and sucrose metabolism” were mainly enriched among the up-regulated DEGs between 0RE+ and 0RE− (Figure 3E). Additionally, “arachidonic acid metabolism”, “biosynthesis of amino acids”, “phenylalanine, tyrosine and tryptophan biosynthesis”, and “carbon metabolism” were enriched in up-regulated DEGs mediated by *E. gansuensis* at 200 mM NaCl stress (Figure 3E). Furthermore, KEGG pathway analysis showed that terms of “plant-pathogen interaction” and “MAPK signaling pathway” were enriched in down-regulated DEGs that were mediated by *E. gansuensis* at both 0 mM and 200 mM NaCl stress (Figure 3F). Secondary metabolic pathways, including “flavone and flavonol biosynthesis”, “monoterpenoid biosynthesis” and “thiamine metabolism”, were repressed by *E. gansuensis* under 0 mM NaCl concentration. Furthermore, pathways involving in “phenylpropanoid biosynthesis”, “taurine and hypotaurine metabolism”, “β-alanine metabolism”, and “galactose metabolism” were mainly enriched in the down-regulated DEGs between 200RE+ and 200RE− (Figure 3F).

### 3.4. Enrichment Analysis of DEGs in Roots of E+ and E− A. Inebrians Regulated by NaCl

**Figure 4:**
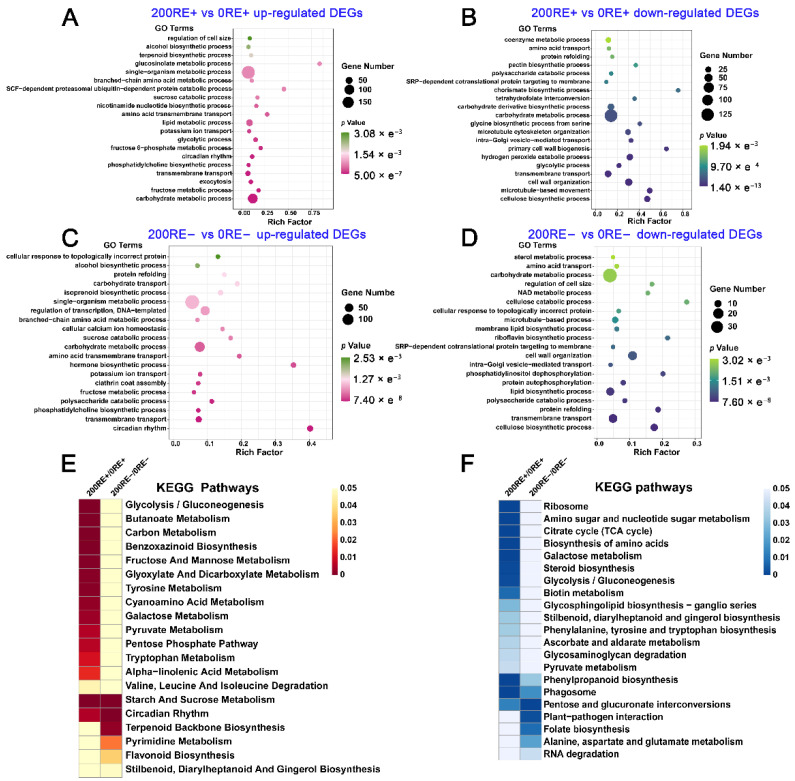
GO terms and KEGG pathway enrichment analysis based on the NaCl-induced DEGs in roots of both E+ and E− *A. inebrians*. The GO terms of up-regulated (**A**) and down-regulated (**B**) DEGs between 200RE+ and 0RE+. The GO terms of up-regulated (**C**) and down-regulated (**D**) DEGs between 200RE− and 0RE−. KEGG pathway enrichment analysis of up-regulated (**E**) and down-regulated (**F**) DEGs between 200RE+/0RE+ and 200RE−/0RE−.

To further characterize the transcriptional changes, enrichment analysis of DEGs in roots of *A. inebrians* regulated by NaCl in the presence and absence of *E. gansuensis* was carried out. Results of GO terms showed that categories of “fructose metabolic process”, “exocytosis”, “phosphatidylcholine biosynthetic process”, “circadian rhythm”, “glycolytic process”, and “potassium ion transport” were significantly enriched in the up-regulated DEGs identified in roots of E+ *A. inebrians* between 0 and 200 mM NaCl concentrations (Figure 4A); in contrast, biological processes involving biosynthesis of cellulose and primary cell wall were enriched in the down-regulated DEGs between 200RE+ and 0RE+ (Figure 4B). We also found that terms of “carbohydrate metabolic process”, “transmembrane transport”, and “glycolytic process” were commonly enriched by the DEGs identified between 200RE+ and 0RE+ (Figure 4A,B). Additionally, categories of “circadian rhythm”, “phosphatidylcholine biosynthetic process”, “fructose metabolic process”, “potassium ion transport”, and “hormone biosynthetic process” were induced by 200 mM NaCl stress in the roots of E− *A. inebrians* (Figure 4C); among the up- and down-regulated DEGs between 200RE− and 0RE−, terms of “transmembrane transport”, “polysaccharide catabolic process”, and “carbohydrate metabolic process” were commonly enriched (Figure 4C,D). Generally, these results suggest that NaCl stress significantly affects physiological processes, including circadian rhythm, potassium ion transport, fructose metabolism, as well as transmembrane transport in roots of E+ and E− *A. inebrians*.

The KEGG pathways enrichment analysis suggested that “starch and sucrose metabolism” and “circadian rhythm” were induced by NaCl stress among DEGs in both RE+ and RE−; whereas terms of “glycolysis”, “benzoxazinoid biosynthesis”, “fructose and mannose metabolism”, “pentose phosphate pathway”, “tryptophan metabolism” and “pyruvate metabolism” were significantly enriched among the up-regulated DEGs between 200RE+ and 0RE+ (Figure 4E); and secondary metabolic pathways involving in the biosynthesis of terpenoid and flavonoid were markedly enriched among the up-regulated DEGs between 200RE− and 0RE− (Figure 4E). In contrast, KEGG analysis showed that the down-regulated DEGs by 200 mM NaCl stress were mainly focused on metabolism processes, including “citrate cycle”, “biosynthesis of amino acids”, “galactose metabolism”, and “steroid biosynthesis” in roots of E+ host plants, and the terms such as “plant-pathogen interaction” and “alanine, aspartate and glutamate metabolism” in roots of E− host plants under 200 mM NaCl stress (Figure 4F). Meanwhile, NaCl stress also repressed the “phenylpropanoid biosynthesis”, “phagosome”, and “pentose and glucuronate interconversions” in roots of both E+ and E− *A. inebrians* (Figure 4F).

### 3.5. E. gansuensis Regulates the Expression of Canonical Genes Associated with Amino Acid Metabolism, MAPK Signaling Pathway, Starch and Sucrose Metabolism, and Circadian Rhythm in Roots of A. inebrians under NaCl Stress

**Figure 5:**
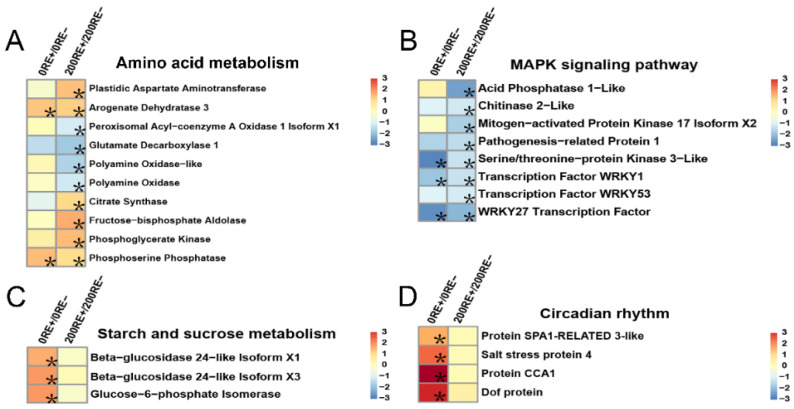
Clustering of DEGs associated with amino acid metabolism (**A**), MAPK signaling pathway (**B**), starch and sucrose metabolism (**C**) and circadian rhythm (**D**) between 0RE+ and 0RE− and between 200RE+ and 200RE−. Color panels stand for log_2_(fold change). Asterisks indicate statistically significant changes with FDR < 0.01 and fold change ≥ 2.

In amino acid metabolism, the infection of *E. gansuensis* induced more DEGs in roots of *A. inebrians* at 200 mM NaCl concentration compared to that at NaCl-free condition (Figure 5A). *E. gansuensis* significantly altered the expression of 10 genes, with up-regulating six genes and down-regulating four genes, of amino acid metabolism under 200 mM NaCl stress. In comparison, at 0 mM NaCl concentration, only two genes, arogenate dehydratase 3 and phosphoserine phosphatase, were remarkably induced by *E. gansuensis* in the host’s roots (Figure 5A). Similarly, more canonical genes involved in the MAPK signaling pathway were significantly repressed by *E. gansuensis* under 200 mM NaCl stress compared to that under 0 mM NaCl, indicating that *E. gansuensis* might inhibit the MAPK signaling as well as restrict the growth of the host’s roots in order to resist the detrimental NaCl stress (Figure 5B). However, *E. gansuensis* did not significantly induce expression of the canonical genes involved in “starch and sucrose metabolism” and “circadian rhythm” under 200 mM NaCl stress. In contrast, transcriptional levels of beta-glucosidase 24-like isoform, glucose-6-phosphate isomerase, and the central circadian clock proteins CCA1 were notably induced by the infection of *E. gansuensis* at 0 mM NaCl concentration [30] (Figure 5C,D).

### 3.6. NaCl Treatment Alters Expression of Main Gene Related to Amino Acid Metabolism, Starch and Sucrose Metabolism, Fructose and Mannose Metabolism, Citrate Cycle, Flavonoid Biosynthesis, and Circadian Rhythm in Roots of E+ and E− Plants

**Figure 6:**
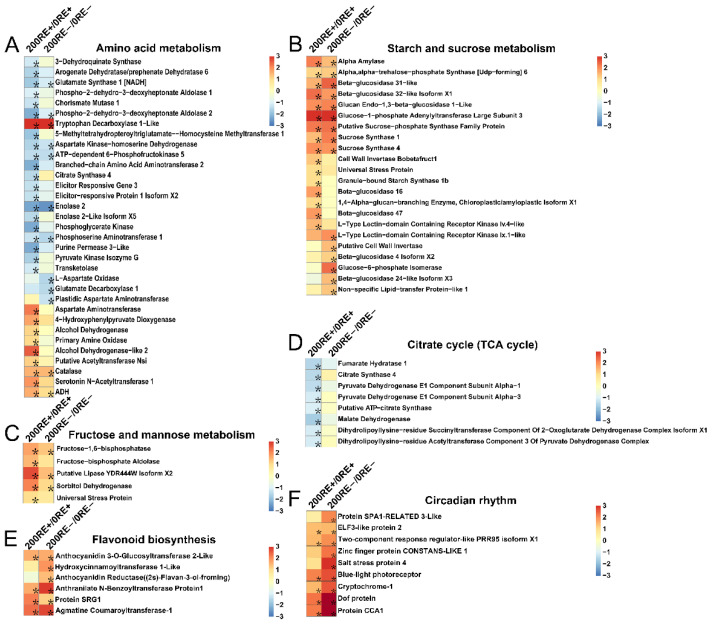
Impact of 200 mM NaCl on amino acid metabolism (**A**), starch and sucrose metabolism (**B**), fructose and mannose metabolism (**C**), citrate cycle (**D**), flavonoid biosynthesis (**E**) and circadian rhythm (**F**) in roots of E+ and E− *A. inebrians*. Color panels stand for log_2_(fold change). Asterisks indicate statistically significant changes with FDR < 0.01 and fold change ≥ 2.

Similarly, in roots of E+ plants, treatment with 200 mM NaCl significantly altered the expression of 30 genes, with the up-regulation of 10 genes and down-regulation of 20 genes of amino acid metabolism in roots of *A. inebrians* (Figure 6A). In contrast, only 12 genes showed significant changes in terms of transcriptional levels between 200RE− and 0RE− (Figure 6A). Additionally, NaCl treatment notably induced the expression of key genes that associated with starch and sucrose metabolism in both E+ and E− host plants (Figure 6B). Likewise, the main genes associated with fructose and mannose metabolism, flavonoid biosynthesis, and circadian rhythm were up-regulated in roots of both E+ and E− plants in response to NaCl treatment (Figure 6C,E,F). Intriguingly, under 200 mM NaCl concentration, infection of *E. gansuensis* inhibited eight genes associated with the citrate cycle in the roots of *A. inebrians*, including the crucial rate-limiting enzyme—citrate synthase 4. However, in the *E. gansuensis*-free plants, expression of these relevant genes did not show any significant alterations, indicating *E. gansuensis* might enhance the resistance of high NaCl levels via repressing the central metabolic pathway in root cells (Figure 6D). 

### 3.7. Effects of E. gansuensis and NaCl on the Metabolic Profiles in the Roots of A. inebrians

The metabolome is a powerful method to study stress biology in plants by identifying the differential compounds, thereby describing an outline of the metabolic situation. Next, metabolome analysis based on GC-MS was used to further investigate the physiological response in roots of *A. inebrians* mediated by *E. gansuensis* as well as NaCl. The score plots of PCA showed a clear separation between 0 and 200 mM NaCl concentrations (Supplementary Materials Figure S4A). On the other hand, the score points for E+ and E− under the same NaCl concentration overlapped, indicating that NaCl treatment played more important roles in changing the metabolomic traits in roots of *A. inebrians* than the presence of endophyte (Supplementary Materials Figure S4A). Additionally, supervised orthogonal partial least squares discriminant analyses (OPLS-DA) showed a clear separation between 0RE+ and 0RE− (Supplementary Materials Figure S4B), between 200RE+ and 200RE− (Supplementary Materials Figure S4C), between 200RE+ and 0RE+ (Supplementary Materials Figure S4D), between 200RE− and 0RE− (Supplementary Materials Figure S4E), indicating there was *E. gansuensis*- or NaCl concentration-dependent metabolic reprogramming in roots of *A. inebrians*.

Next, significantly different metabolites were identified between the E+ and E− plants under 0 and 200 mM NaCl concentrations. Compared to the E− plants, six metabolites, including lysine, salicin, noradrenaline, 4-aminobutyric acid, acetol, and aconitic acid, exhibited a significant increase in roots of E+ plant under 0 mM NaCl concentration (Supplementary Materials Figure S5A). In contrast, three kinds of alcohols and two kinds of carbohydrates were identified between RE+ and RE− in response to the 200 mM NaCl treatment (Supplementary Materials Figure S5B). Additionally, among these metabolites, galactinol, a kind of desiccation-associated sugar, exhibits the most significant increase with 18.55-fold changes (Supplementary Materials Table S2). Intriguingly, compared to *E. gansuensis*, NaCl was able to alter the metabolites to a larger extent. Meanwhile, 27 and 33 differentially expressed metabolites were found in the roots of E+ and E− plants in response to 200 mM NaCl treatment, respectively (Supplementary Materials Figure S5C and S5D). Roots of E+ plant treated with 200 mM NaCl exhibited negative effects on the metabolic profiles that associated with organic acids, alcohols, aldehydes, and ketones compared to 0 mM NaCl treatment (Supplementary Materials Figure S5C). On the contrary, NaCl only markedly improved the content of 2-monopalmitin (19.15-fold) and leucine (1.79-fold) in roots of E+ plants compared to the NaCl-free treatment (Supplementary Materials Figure S5C, Table S2). Our results also suggested that NaCl treatment significantly impaired the content of organic acids (such as 6-phosphogluconic acid, mucic acid, chlorogenic acid, oleic acid, D-glyceric acid, orotic acid), sugars (such as raffinose, maltotriose, melezitose, trehalose), ketones, alcohols and esters in roots of E− plants compared to NaCl-free condition (Supplementary Materials Figure S5D). Meanwhile, 200 mM NaCl showed significantly positive effects on the levels of trehalose (4.16-fold), tryptophan (4.1-fold), leucine (2.48-fold), pipecolinic acid (3.06-fold), guanosine (16.94-fold), glycolic acid (2.23-fold) in E- roots compared to 0 mM NaCl treatment (Supplementary Materials Figure S5D and Table S2).

### 3.8. Effects of E. gansuensis on Metabolic Pathways in the Roots of A. inebrians under 0 and 200 mM NaCl Concentrations

**Figure 7:**
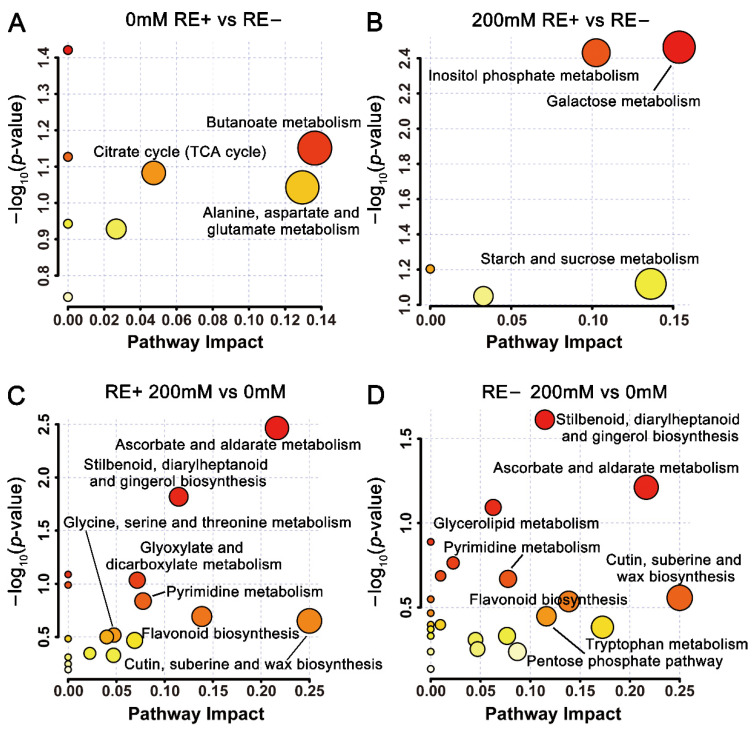
Bubble plots showing the enrichment analysis of altered metabolic pathways in roots. (**A**,**B**) represent the differential pathways between RE+ and RE− under 0 mM and 200 mM NaCl concentrations, respectively. (**C**,**D**) represent the differential pathways between 200 mM and 0 mM NaCl treatment in RE+ and RE-, respectively. The plots were organized by pathway enrichment analysis (log_10_(*p*-values)) and pathway topology analysis (pathway impact).

Furthermore, metabolic pathways were established based on the differently expressed metabolites that were identified between RE+ and RE− under 0 and 200 mM NaCl concentrations, respectively. As shown in Figure 7A, infection of *E. gansuensis* most probably altered the metabolic pathways associated with butanoate metabolism, alanine, aspartate and glutamate metabolism, and TCA cycle under NaCl-free conditions. Under 200 mM NaCl concentration, the main pathways involved in inositol phosphate metabolism, galactose metabolism, starch and sucrose metabolism were enriched between RE+ and RE− (Figure 7B). It is also suggested that ascorbate and aldarate metabolism, stilbenoid, diarylheptanoid and gingerol biosynthesis, glyoxylate and dicarboxylate metabolism, pyrimidine metabolism, flavonoid biosynthesis, cutin, suberine, and wax biosynthesis were the most correlative pathways mediated by 200 mM NaCl in roots of E+ plants (Figure 7C). Similarly, stilbenoid, diarylheptanoid and gingerol biosynthesis, ascorbate and aldarate metabolism, glycerolipid metabolism, pyrimidine metabolism, cutin, suberine, and wax biosynthesis, flavonoid biosynthesis, tryptophan metabolism, and pentose phosphate pathway were the main metabolic pathways that identified between 200RE− and 0RE− (Figure 7D). Intriguingly, there were 10 metabolic pathways that shared between 0 and 200 mM NaCl treatment in E+ and E- roots, accounting for 66.7% of pathways found in 200RE+ vs. 0RE+ and for 90.9% of pathways in 200RE− vs. 0RE− (Supplementary Materials Figure S6), and we found that NaCl specifically regulated the main metabolic pathways involving in “galactose metabolism”, “glycerophospholipid metabolism”, “pentose phosphate pathway”, “starch and sucrose metabolism”, and “valine, leucine and isoleucine degradation” in roots of E+ plants. In contrast, the TCA cycle was the only differential metabolic pathway that specifically enriched between 200RE− and 0RE− (Supplementary Materials Figure S6).

## 4. Discussion

Soil salinization is one of the important factors that severely affect plant growth and productivity and has become a worldwide threat to the sustainable development of both agriculture and stock farming. Plants of *A. inebrians* with infection of *E. gansuensis* exhibit greater resistance to salt stress than *E. gansuensis*-free plants [6,20]. Our results have suggested that the presence of *Epichloë* endophytes in leaves altered multiple biological processes, including photosynthesis, oxidation-reduction, amino acid metabolism, and flavonoid biosynthetic, to enhance the adaptation of host plants to NaCl stress [7]. Despite the current knowledge, information referring to the underlying molecular mechanism in roots regulated by *Epichloë gansuensis* under salt stress is still elusive. In the current study, our results showed that the aerial *E. gansuensis* reprogrammed the transcriptional events and altered metabolic levels in the roots of host plants to gain better resistance under NaCl stress.

### 4.1. E. gansuensis, an Important Factor for the Transcriptional Reprogramming in Roots of A. inebrians

*E. gansuensis* significantly regulated up to 21.1% of the total identified transcripts of host *A. inebrians* to achieve the better trade-off between the host’s growth and salt resistance. The analogous tactics have been reported by other studies. Dupont et al. found that infection of *E. festucae* resulted in dramatic alterations in the expression of more than 38% of ryegrass genes, favoring host genes involved in the secondary metabolism [22]. Moreover, we previously reported that *E. gansuensis* significantly altered massive genes in leaves of *A. inebrians* in response to NaCl stress [7]. In contrast, relatively few genes with significantly altered expression were identified in tall fescue due to the presence of *E. coenophiala*, and the mainly annotated DEGs were involved in defense and abiotic response [31]. Furthermore, the whole-transcriptome analysis revealed only 0.5% (256/50,000) of the host rice genes exhibited clear changes in expression level upon the infection of *G. intraradices* [32]. Meanwhile, some studies suggested that only a small percentage (1–3%) of host genes were significantly affected by the fungal colonization, such as *Medicago truncatula* interacting with *Glomus intraradices* [33], tomato with *Funneliformis mosseae* [34], and rice with *Piriformospora indica* [35]. In fact, different host plants and endophyte or sequencing techniques might account for the differences in the number of DEGs revealed by different reports [7,31].

### 4.2. The Regulation of Energy Metabolism Acts as a Crucial Strategy for Salt Tolerance of Host’s Roots Mediated by E. gansuensis

**Figure 8:**
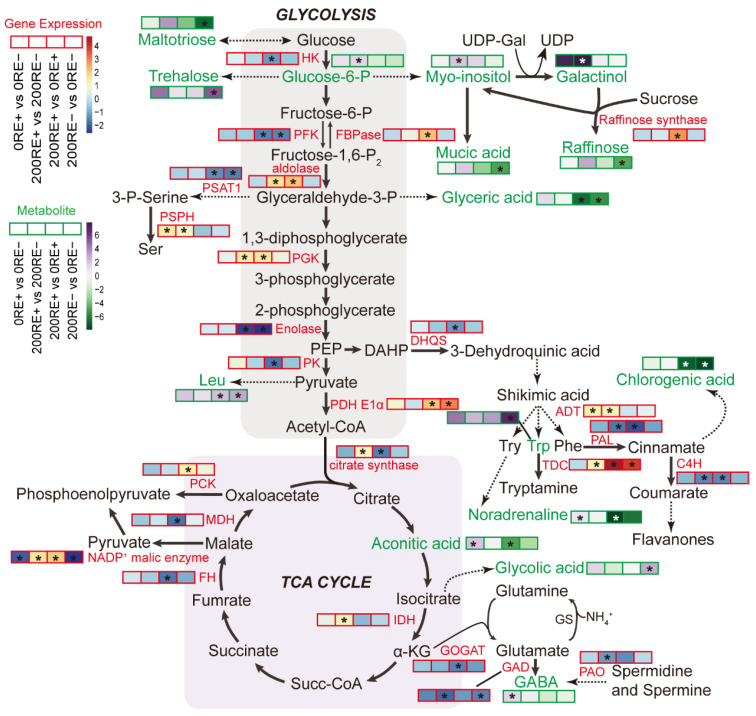
Integration of changes in transcription and metabolite levels mapped to the central metabolic pathways in roots of E+ and E− *A. inebrians* at 0 and 200 mM NaCl concentrations. Boxes with red and green frames denote the gene expression and metabolites, respectively. The straight arrows represent direct paths, and the dotted arrows represent indirect paths. Asterisks represent statistically significant changes (*p* ≤ 0.05).

The subtle control of energy-releasing reaction plays a vital role in the accommodation of plants to abiotic stress [36]. In our results, as depicted in Figure 8, the main energy metabolism pathways, including glycolysis, TCA cycle, raffinose, and related carbohydrate metabolism, have emerged in this integration mode. In this mode, a lot of genes showed significant alteration due to the infection of *E. gansuensis* under salt stress, indicating that the reprogramming of gene expression in the central metabolic pathways was crucial for the salt-adaption of E+ *A. inebrians*. Analysis of Metabolomics is an effective complement to transcriptomics at the metabolite level. In this mode, we find that there is a gap between metabolic adjustments and transcriptional regulation, consistent with the pattern in yeast as described by Hackett et al. [37]. The possible reasons for this gap are as following: (1) the inherent limitation of GC-MS determines that only low molecular weight (<600 Da) and volatile compounds can be separated and identified [38], therefore, some metabolites with polar and high molecular weight were absent from the mode; (2) Other regulatory modes except gene expression regulatory are involved in the generation of metabolites, such as phosphorylation and dephosphorylation regulation; (3) feedback regulation from the physiological to the genetic level.

The TCA cycle connects energy generation with C and N metabolism, and it is tightly regulated by the adaptative changes of mitochondria under various stresses such as salt injury [39]. Salt stress unavoidably alters the TCA cycle, and it has been reported in diverse plants such as tobacco [40], grapevine [41], and Arabidopsis [42]. Salinity can also cause a contrasting effect on the enzyme activities of the TCA cycle in two rice cultivars, Nonabokra and MTU 1010 [39]. Furthermore, the level of TCA cycle intermediates exhibited an increase in salt-tolerant barley Sahara but remained non-altered in the NaCl-sensitive cultivar Clipper [43]. In our results, interestingly, the activity of TCA cycle in E+ roots was lower at both transcription and metabolite levels under salt stress, indicating a decrease in the flux of carbohydrates from glycolysis metabolism. A decrease in plant respiration represents a long-term response under salt stress, and this coincides with lower reliance on energy metabolism as growth is slowed [43]. Together, these results suggested that infection of *E. gansuensis* might help *A. inebrians* to quickly adapt to the adverse salinity condition.

Organic acids, mainly synthesizing in mitochondria via the TCA cycle, are of importance in energy production, amino acid biosynthesis, osmotic adjustment, and balance of cation excess at the cellular level [44]. It has been reported that the total organic acid content in the roots of alfalfa was decreased by more than 40% after salt treatment [45]. Consistent with the previous study, our metabolic data showed that most of the organic acids were depressed in the roots of E+ and E− plants after the application of 200 mM NaCl solution (Figure S5C and S5D). Under NaCl stress, *A. inebrians* could restrain the synthesis of organic compounds in order to decrease energy consumption. This special strategy to adapt stresses has also been reported in sunflowers [46]. Furthermore, we found that infection of *E. gansuensis* induced the accumulation of 4-aminobutyric acid and aconitic acid in the roots of host plants. The 4-aminobutyric acid usually exhibits potential functions in carbon metabolism, plant development and defense, nitrogen storage, and pH regulation [47]. Aconitic acid plays various biological functions in plant cells as an inflammatory inhibitor or an antifeedant [48]. *E. gansuensis* might improve the competitiveness of host plants by mediating the accumulation of 4-aminobutyric acid as well as aconitic acid under normal growth conditions.

### 4.3. The Roles of Amino Acid and Nitrogen Metabolism in Salt-Resistance of Roots of A. inebrians

It has been suggested that reorganization in amino acid metabolism is required for plants to cope with diverse abiotic stress [49]. Aromatic amino acids are synthesized via the shikimate pathway in plants and serve as precursors for the biosynthesis of proteins as well as diverse secondary metabolites that have a positive effect on the plant’s resistance to biotic and abiotic stress [50,51]. Our transcriptome results showed that infection of *E. gansuensis* significantly induced the phenylalanine, tyrosine, and tryptophan biosynthesis in roots of *A. inebrians* under 200 mM NaCl concentration compared to the endophyte-free plants (Figure 3E). Meanwhile, pathways involved in tyrosine metabolism and tryptophan metabolism were markedly enriched in roots of E+ plants after treatment with 200 mM NaCl (Figure 4E), but these pathways were not identified in E− plants, indicating the crucial role of *E. gansuensis* in the regulation of phenolic amino acid metabolism in NaCl-treated plants. Additionally, differently expressed metabolites identified by GC-MS also supported the important roles of amino acid metabolism for *A. inebrians* in response to NaCl stress. We found that NaCl treatment significantly altered the metabolic pathways, such as glycine, serine, and threonine metabolism and tryptophan metabolism, in roots of E+ and E− plants (Figure 7C,D). Similarly, gene expression involved in glycine, serine, and threonine metabolism was significantly induced in tomato after NaCl treatment, but its regulatory mechanisms for salt adaptation still need to be further explored [52]. Lysine plays an important role in protecting plants from pathogen attacks, and it induces the production of pipecolic acid during the synthesis of salicylic acid (SA) [53]. The infection of endophytes might enhance tolerance to the pathogen invasion in host plants as lysine was significantly induced in the roots of E+ plants (Supplementary Materials Figure S5A).

The nitrogen metabolic process plays an important role in the adaption of plants to salinity resistance [6]. Glutamine synthetase (GS) involves in nitrogen metabolism as well as proline synthesis in plant cells, and transgenic rice with overexpressed *GS* showed an enhancement in resistance to salt stress [54]. However, our results suggested that NaCl treatment repressed the expression of the *GS* gene in the roots of both E+ and E− plants. These results were contrary to the previous findings that endophyte enhanced the *GS* expression of host leaves in response to NaCl stress [7] and that GS activity was higher in leaves and roots of endophyte-infected plants than that of endophyte-free plants under 200 mM NaCl concentration [6]. The differences might result from different tissues used for analysis and posttranscriptional modification for GS in roots [55,56]. Moreover, our result also showed that the transcriptional levels related to aromatic amino acid biosynthesis, the Asp-derived amino acid pathway, and the phosphorylated pathway of serine biosynthesis were down-regulated in both E+ and E− plants under NaCl stress. Aspartate aminotransferase (Asp) catalyzes the reversible transamination of aspartate into glutamate and plays a key role in the carbon and nitrogen distribution of plants [57]. It has been reported that salt and drought stress up-regulated the expression of *Asp3* in Arabidopsis [58]. The 4-hydroxyphenylpyruvate dioxygenase (HPPD) participates in the biosynthesis of vitamin E, a kind of compound that functions as a lipophilic antioxidant to maintain membrane integrity. Kim et al. indicated that overexpression of *HPPD* enhanced the resistance of transgenic plant sweet potatoes to diverse abiotic stress, including drought, salt, and oxidative stresses [59]. In our study, we found that *E. gansuensis* significantly induced both expression of genes *Asp* and *HPPD* in roots of *A. inebrians* under 200 mM NaCl concentration, but these two genes were not identified among the DEGs between 200RE− and 0RE−, indicating that endophyte might specifically up-regulate the *Asp* and *HPPD* genes to enhance the adaption of *A. inebrians* to salt treatment.

### 4.4. Physiological Response of Carbohydrate and Lipid Metabolism Underlying Salt Tolerance in E. gansuensis-infected A. inebrians

In addition to ionic and oxidative stress, high salt concentration also result in adverse osmotic stress in plant cells; thus, osmotic regulation is critical for plant adaptation under salt stress. Though free amino acids and amides have been widely reported to accumulate in plant cells exposed to salt stress and play a role in osmotic adjustment [60], the root of endophyte-infected *A. inebrians* appeared to preferentially regulate the carbohydrate metabolism to accommodate the improper osmotic pressure caused by high salt concentration. Our results suggested that *E. gansuensis* altered the expression of genes associated with carbohydrate metabolism, glucose metabolism, and fructose metabolism and induced the fructose 2,6-bisphosphate metabolism but repressed the polysaccharide catabolic process under 200 mM NaCl concentration. It has been indicated that the root-colonized *P. indica* altered carbohydrate metabolism to enhance the systemic salt tolerance in the leaf tissue of barley under NaCl treatment [61], which is consistent with our results. Li et al. indicated that the beneficial colonization of *B. cereus* remarkably affected the tobacco carbohydrate metabolism by inducing the expression of one β-amylase, two 1,4 α-glucan branching enzymes, three α-amylase, and three iso-amylase, and down-regulating another three β-amylase [62]. In our results, 200 mM NaCl treatment significantly induced the key enzymes involved in starch and sucrose metabolism, such as sucrose synthases, α-amylase, β-glucosidase, glucan endo-1,3-β-glucosidase and putative sucrose-phosphate synthase family protein in the root of E+ and E− plant (Figure 6B), indicating endophyte reprogrammed the carbohydrate metabolism of the host plant to better accommodate to the salt stress.

Reports about salt-induced changes in the lipid composition of cells have been presented in diverse plant species, such as halophytes and glycophytes. Alterations in membrane lipids exhibit a direct influence on the activity of membrane proteins or signaling molecules, the membrane integrity and permeability, as well as signaling pathways mediated by lipids [63]. It has been reported that the colonization of a salt-tolerant endophyte *Fusarium sp*. confers the resistance of host rice IR-64 to salt stress, which is related to the altered expression of metabolism networks, including secondary metabolism, lipid metabolism, and redox metabolism [64]. In the plasma membrane, sterols are lipid composition of microdomain, namely lipid rafts, which involves in a lot of cellular processes, including regulation of the activity of Na^+^(K^+^)-ATPase in plant cells [65,66]. Meanwhile, lipids such as steroids, phosphatidylcholine, phospholipid, and sphingolipid may serve as lipid signaling that is able to enhance the resistance to various abiotic stress [67]. In the current study, we found that the infection of *E. gansuensis* altered the lipid biosynthetic process. Additionally, *E. gansuensis* up-regulated the gene expression of phosphatidylinositol dephosphorylation but down-regulated the expression of genes associated with cellular lipid metabolic process, long-chain fatty acid metabolic process, lipid biosynthetic process, and phosphatidylcholine biosynthetic process under 200 mM NaCl concentration, indicating intricate lipid metabolic processes were reprogrammed in roots of *A. inebrians* mediated by *E. gansuensis* in order to improve the adaptation of host plants to salt stress.

### 4.5. E. gansuensis Regulates the Secondary Metabolism and Circadian Rhythm of A. inebrians Roots in Response to Salt Stress

Secondary metabolites can act as a group of bioactive molecules that are responsible for natural biostimulant activities and may confer plant protection against various environmental stresses [68]. The fungal endophytes are usually regarded as important factors in triggering the reprogramming of secondary metabolism in host plants [14]. It has been reported that biological processes, including “biosynthesis of secondary metabolites”, were significantly enriched among the DEGs identified between *P. indica*-colonized rice and endophyte-free rice, and the flavonoid biosynthetic process of host plants was activated in response to the infection of *P. indica* during high salinity stress [35]. Flavonoids, a large group of phenolic compounds, are commonly regarded as antioxidative agents and play conspicuous roles in enhancing plant tolerance to abiotic stress [69]. In our results, we found that genes associated with flavonoid biosynthesis were generally up-regulated by 200 mM NaCl treatment in *E. gansuensis*-free host plants. However, the infection of *E. gansuensis* appeared to weaken the transcriptional alteration that was identified between 200RE− and 0RE−. The probable reason might be that strategy of flavonoid metabolism was preferentially used in endophyte-free *A. inebrians* but was possibly downgraded due to the colonization of *E. gansuensis* as E+ plants might establish better tactics, such as regulation of circadian rhythm, to protect against salt stress. Additionally, it has been reported that salt stress increases the expression of multifunctional terpenoid synthases in mangroves, and terpenoids might play a protective role in resisting high salt concentration [70]. In our present study, we found that the terpenoid biosynthetic process was also significantly enriched among the up-regulated DEGs identified between 200RE+ and 0RE+. In summary, our results suggested that the infection of *E. gansuensis* was able to change the secondary metabolism processes of roots to improve the accommodation of host plants to NaCl stress.

The finely tuned circadian rhythm is essential for plants to acclimate successfully to abiotic and biotic stress [71]. Various biological processes, including the biosynthesis and turnover of stress hormone ABA, cytosolic Ca^2+^ oscillations, and extensive salt-regulated or -responsive genes, are gated by the circadian clock [72]. Melatonin, an important regulator of circadian rhythm, could alleviate the detrimental effect of high salinity on diverse plant species via scavenging salinity-induced ROS, promoting plant photosynthesis, and activating polyamine metabolism pathway [73]. In plants, tryptophan decarboxylase (TDC) and serotonin N-acetyltransferase (SNAT) participated in the melatonin biosynthesis as well as resistance to NaCl stress [74,75]. Consistent with these reports, we found NaCl significantly up-regulated both genes *SNAT* and *TDC 1* in E+ plants. Additionally, in this study, we observed that the circadian rhythm pathway was significantly enriched among the up-regulated DEGs in the roots of E+ *A. inebrians* at 0 mM and 200 mM NaCl, indicating that *E. gansuensis* might enhance the resistance of host plants to salt stress via adjusting the intrinsic circadian rhythm. Similarly, compared to the arbuscular mycorrhiza fungi (AMF)-free *Asparagus officinalis*, the circadian rhythm pathway in leaves of *A. officinalis* was also significantly induced by AMF under salinity stress [76], indicating that regulation of circadian rhythm might commonly act as an effective approach for plant-endophyte symbiosis to the positive response to salt stress.

## 5. Conclusions

In summary, the current study provided new insights into the potential molecular mechanism that *E. gansuensis* improves the resistance of *A. inebrians* to salt stress via exploring the transcriptome and metabolome of the host’s roots. The results suggested that the above-ground *E. gansuensis* were able to act as a crucial factor in regulating the expression of genes, and as much as one-fifth of the identified genes exhibited significantly different expression as the infection of the *E. gansuensis* under NaCl stress. *E. gansuensis* regulated the biological processes involved in carbohydrate metabolism, lipid metabolism, glycolysis, TCA cycle, amino acid metabolism, secondary metabolism, as well as circadian rhythm in the roots of host plants. In addition, *E. gansuensis* also modulated the metabolic profiles of the host’s roots by repressing the levels of organic acids, alcohols, aldehydes, and ketones and increasing the content of leucine to improve the accommodation to salt stress. Under the background of global soil degradation resulting from salinization, these findings provide new avenues to produce salt-tolerant crops or forage by using the endophyte in the future.

## Data Availability

Data are contained within the article.

## Supplementary Materials

The following supporting information can be downloaded at: https://www.mdpi.com/article/10.3390/jof8101092/s1, Figure S1: Heat map matrix showing the Pearson correlation among samples, Figure S2: Volcano plots of differently expressed genes, Figure S3: Heatmap exhibits hierarchical clustering of differentially expressed genes, Figure S4: The principal component analysis (PCA) and orthogonal partial least squares discriminant analysis (OPLS-DA) for metabolic profiles, Figure S5: Histogram for the significantly different metabolites, Figure S6: Venn diagram analysis of differential metabolic pathways identified between 200 mM and 0 mM NaCl concentration in roots of E+ and E− plants; Table S1: Statistical table of Illumina sequencing output and alignment results between sample sequencing data and PacBio data, Table S2: Concentrations for significantly changed metabolites identified between E+ and E− plants under 0 and 200 mM NaCl treatment.
