# Supplementary material for: Comprehensive Analysis of Transcriptome and Metabolome Elucidates the Molecular Regulatory Mechanism of Salt Resistance in Roots of Achnatherum inebrians Mediated by Epichloë gansuensis
Source: J Fungi (Basel). 2022 Oct 17;8(10):1092. doi: 10.3390/jof8101092 (PMC9605608; doi:10.3390/jof8101092)
Supplement: Supplementary file 1 [file jof-08-01092-s001.zip › jof-1830023-supplementary.pdf]

Supplement Data

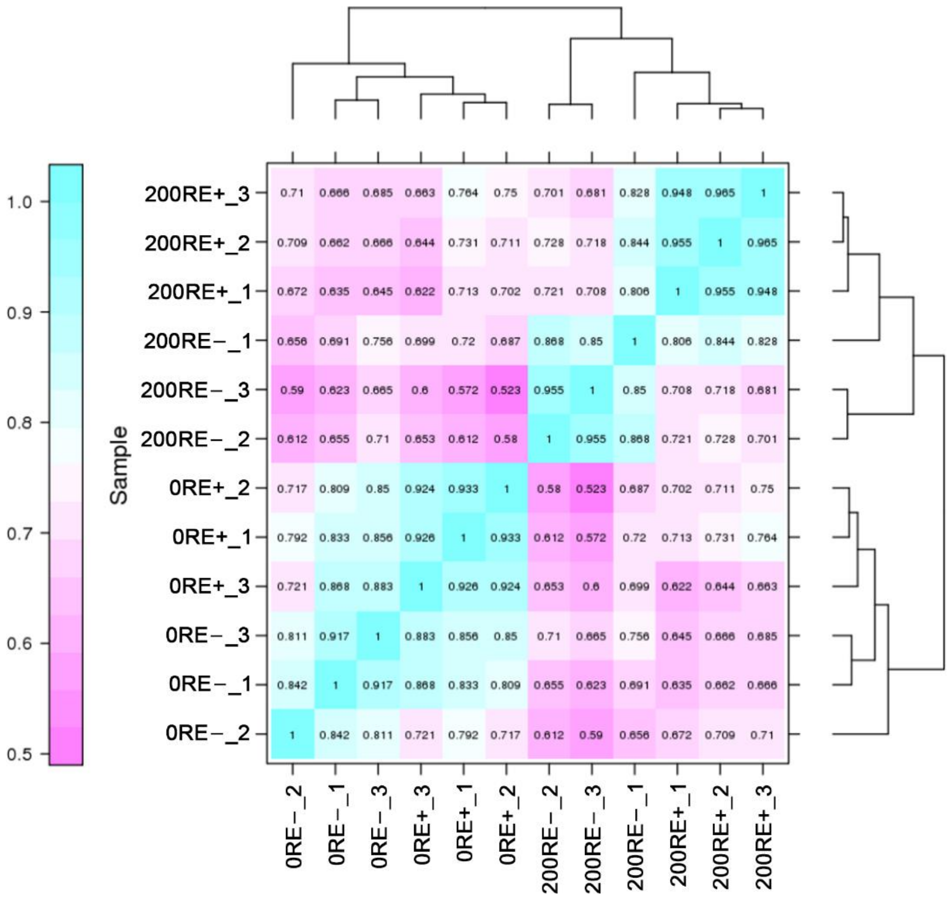

Supplement Figure S1. Heat map matrix showing the Pearson correlation among samples. Color bar represents the correlation coefficient (r^2).

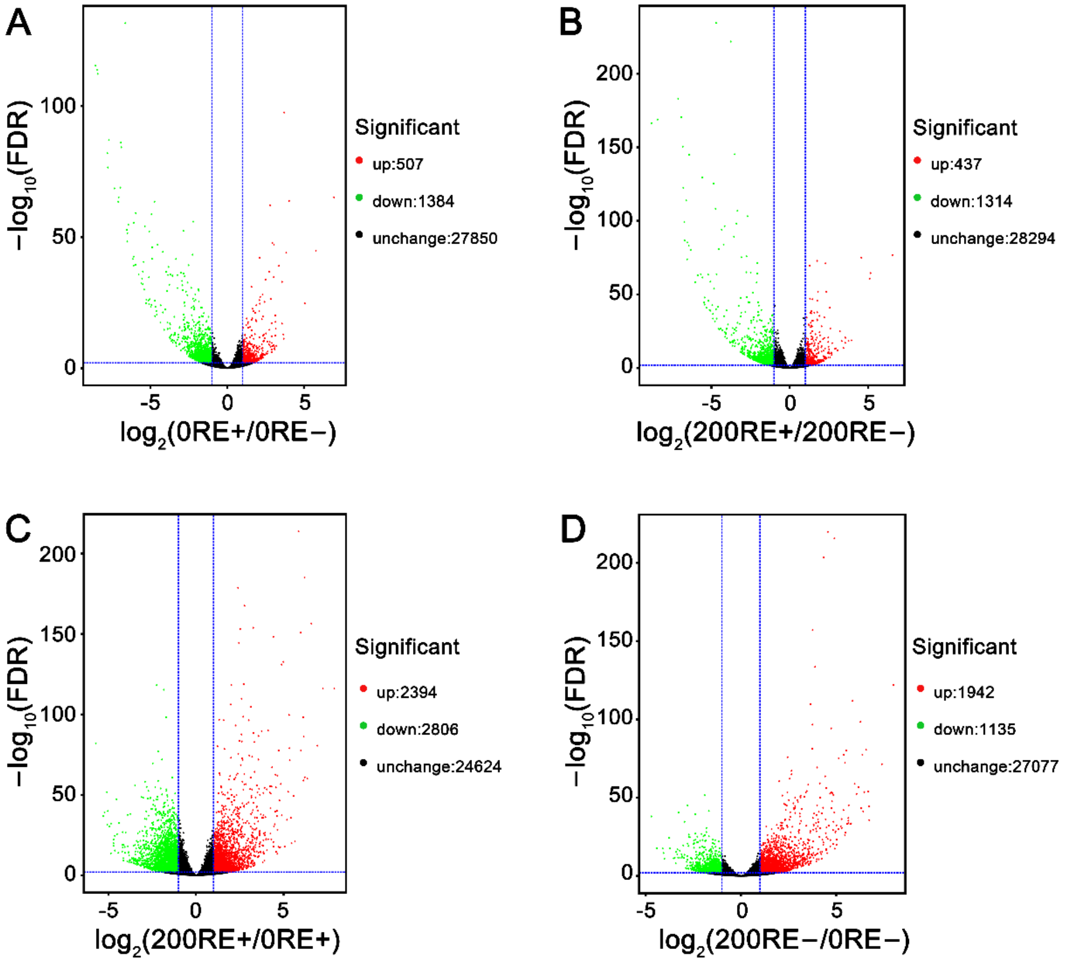

Supplement Figure S2. Volcano plots of differently expressed genes for 0RE+ vs 0RE− (A), 200RE+ vs 200RE− (B), 200RE+ vs 0RE+ (C) and 200RE− vs 0RE− (D). The data for all identified genes are plotted as $\log_2$(fold change) versus the $-\log_{10}$(FDR). Red dots represent the up-regulated DEGs, green dots represent the down-regulated DEGs, and black dots represent the genes with unchanged expression.

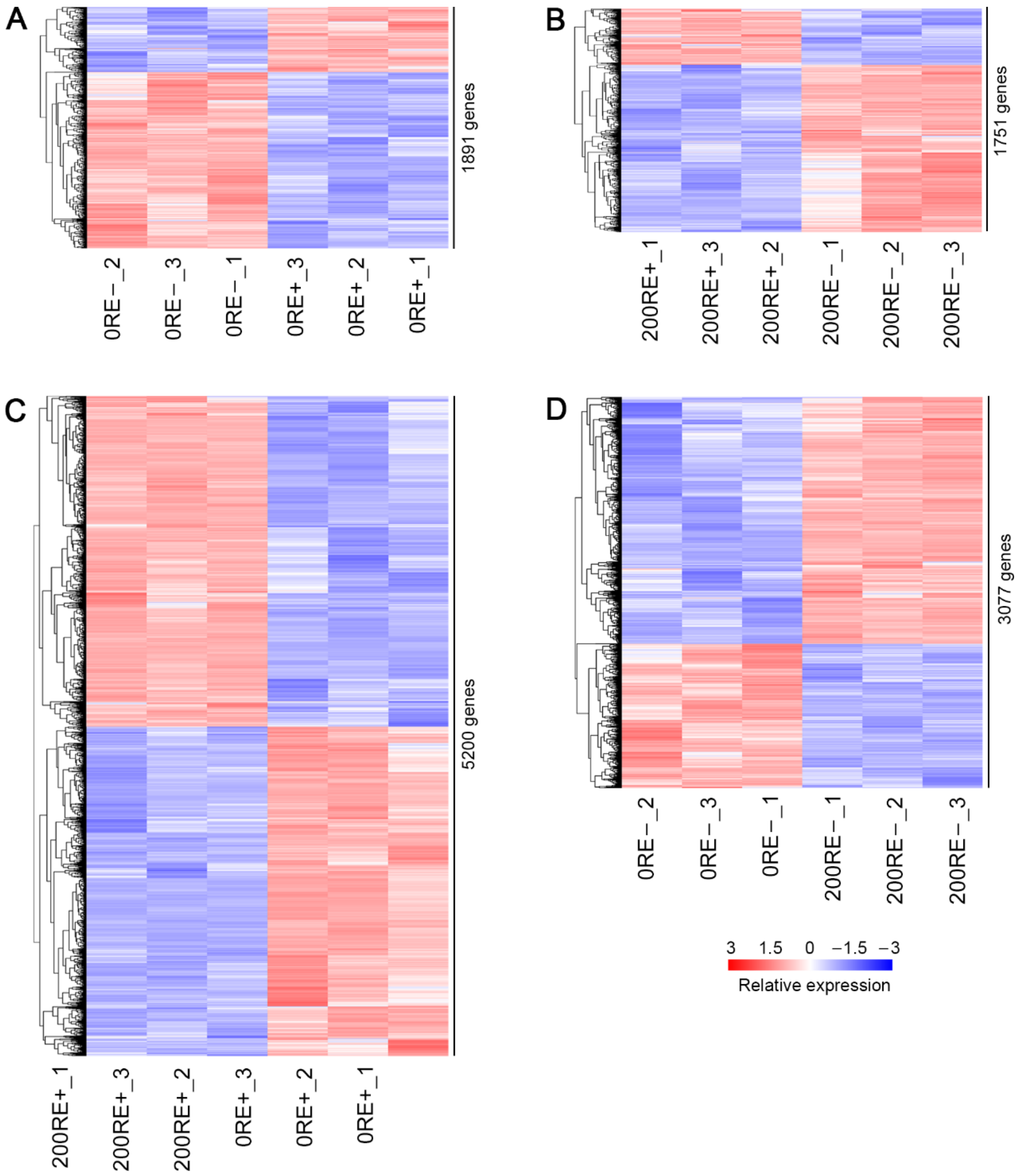

Supplement Figure S3: Heatmap exhibits hierarchical clustering of differentially expressed genes (DEGs) identified in comparative groups of "0RE+ vs 0RE−" (A), "200RE+ vs 200RE−" (B), "200RE+ vs 0RE+" (C), and "0RE− vs 200RE−" (D). Values are row-scaled to show relative expression. Blue and red represent low and high levels respectively. The relative expression is calculated using formula $\log_2$(fold-change).

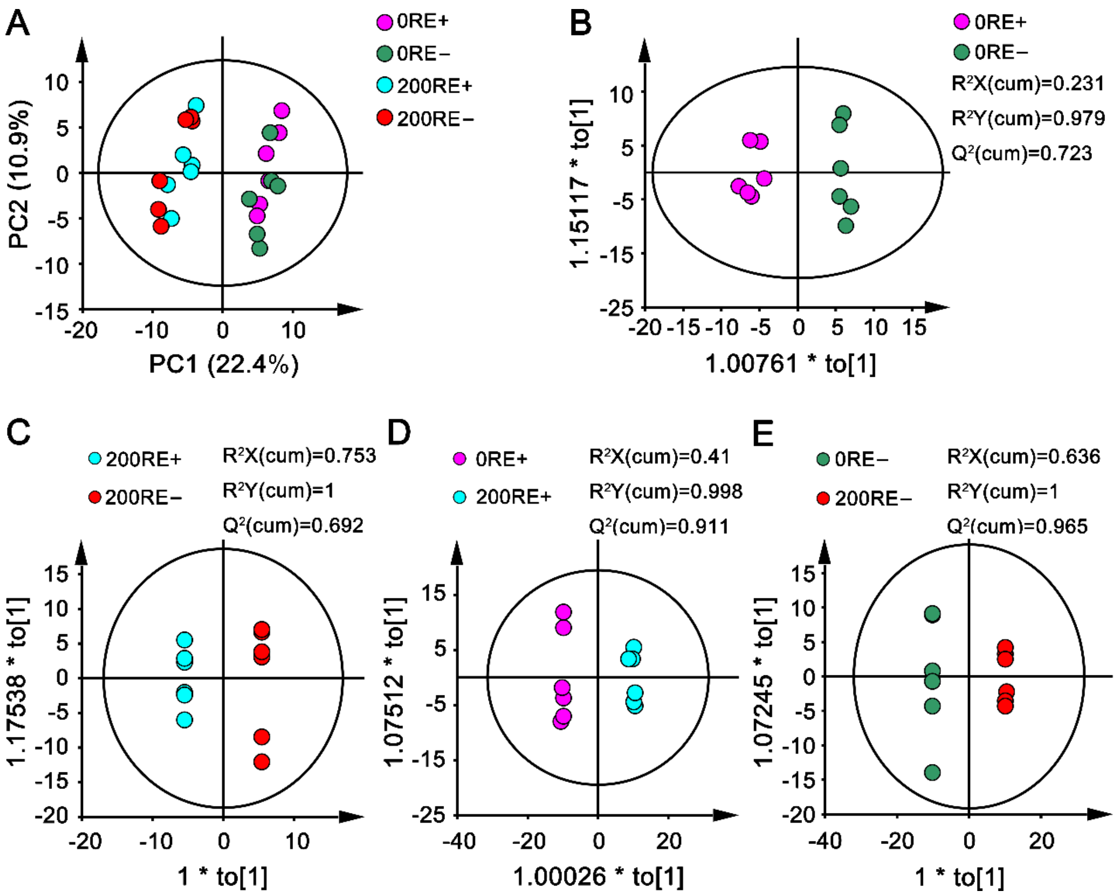

Supplement Figure S4. The principal component analysis (PCA) for metabolic profiles of roots of E+ and E− plants under 0 mM and 200 mM NaCl concentration (A). And the score plots of orthogonal partial least squares discriminant analysis (OPLS-DA) exhibited the endophyte-dependent metabolite difference between RE+ and RE− under NaCl-free condition (B), between RE+ and RE− under 200 mM NaCl concentration; and the NaCl dose-dependent metabolite difference between 200 mM and 0 mM NaCl in RE+ (D), between 200 mM and 0 mM NaCl in RE− (E).

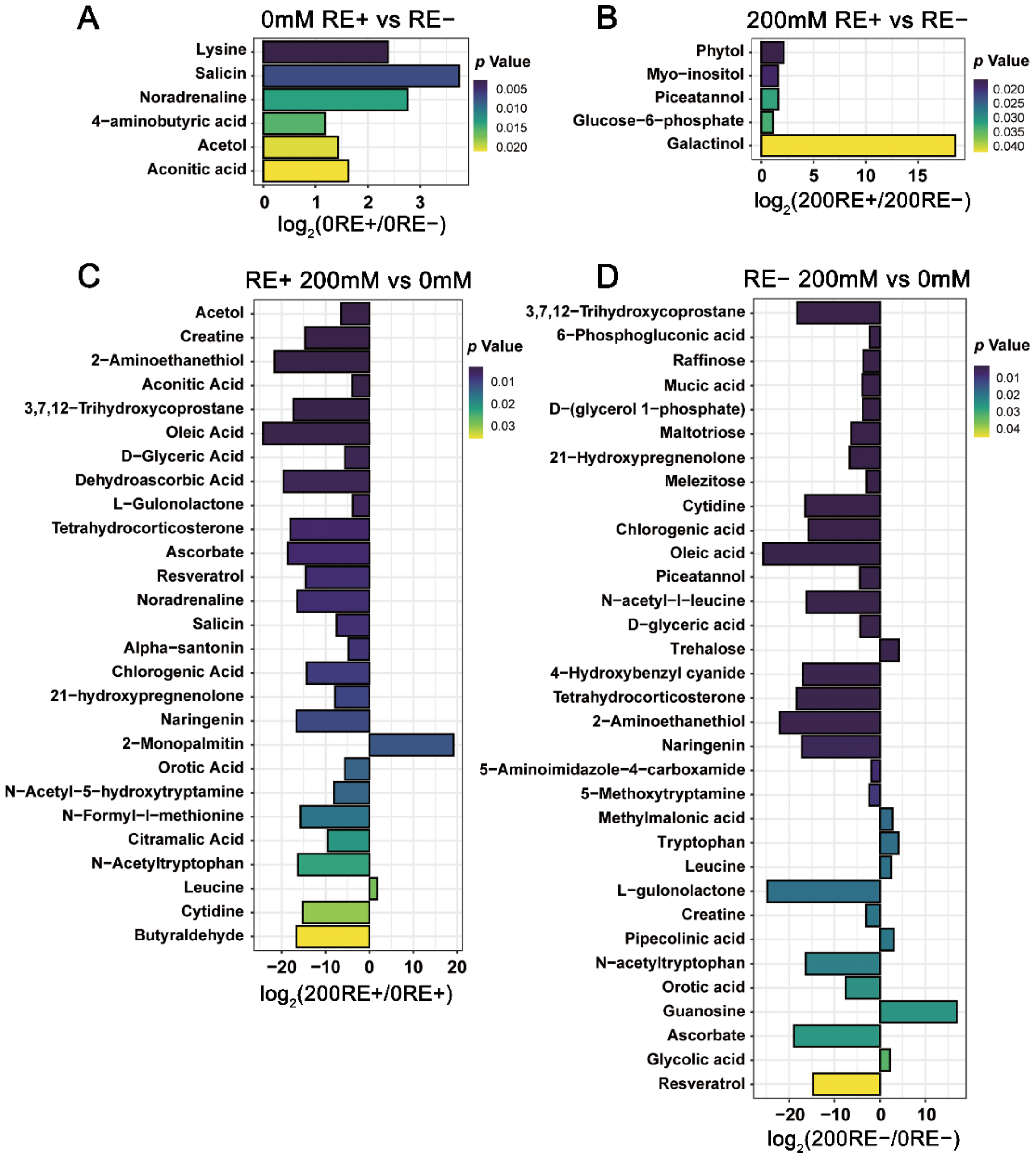

Supplement Figure S5. Histogram with fold changes and *p*-value showed the significantly different metabolites between RE+ and RE− at 0 mM (A) and 200 mM NaCl concentration (B); and between 200 mM and 0 mM NaCl treatment in RE+ (C) and RE− (D). Fold changes were evaluated with the formula log_2(RE+/RE−) and log_2(200 mM/0 mM).

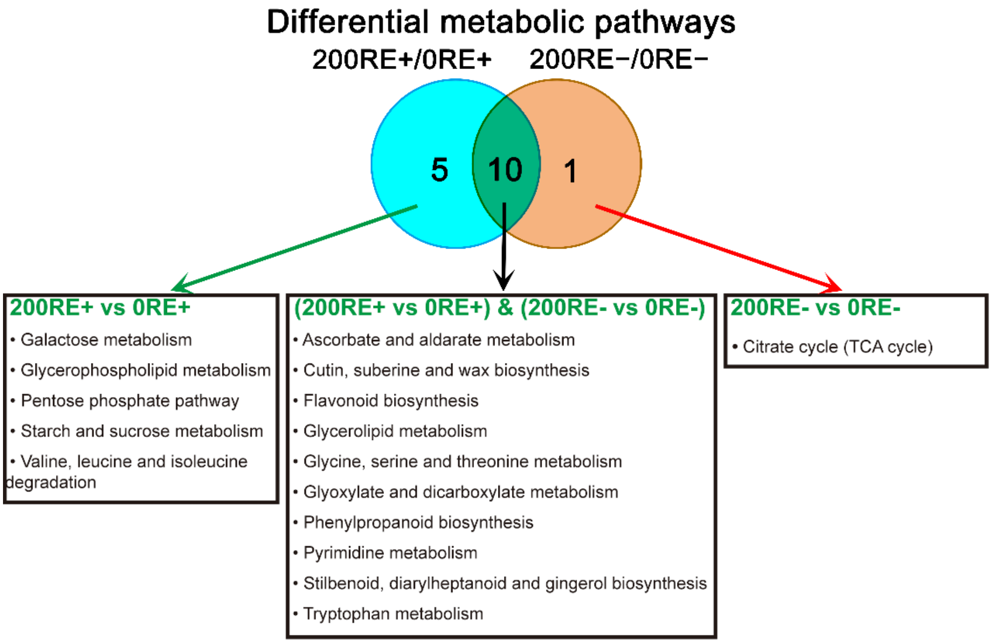

Supplement Figure S6. Venn diagram analysis of differential metabolic pathways identified between 200 mM and 0 mM NaCl concentration in roots of E+ and E− plants. The overlapping region represents pathways shared in the two comparative groups, and the remaining regions indicate differential pathways in the corresponding groups.

Supplement Table S1. Statistical table of Illumina sequencing output and alignment results between sample sequencing data and PacBio data.

Samples	Clean reads	% ≥ Q30	Total reads	Mapped reads
0RE+_1	21,856,136	94.55%	43,712,272	31,699,873 (72.52%)
0RE+_2	21,358,666	94.42%	42,717,332	32,429,373 (75.92%)
0RE+_3	22,432,593	94.45%	44,865,186	35,042,735 (78.11%)
0RE−_1	19,085,883	94.28%	38,171,766	28,704,388 (75.20%)
0RE−_2	22,272,858	94.95%	44,545,716	30,790,736 (69.12%)
0RE−_3	19,230,555	94.71%	38,461,110	29,868,599 (77.66%)
200RE+_1	21,881,327	94.40%	43,762,654	32,996,379 (75.40%)
200RE+_2	22,296,357	94.71%	44,592,714	33,634,593 (75.43%)
200RE+_3	22,225,156	94.55%	44,450,312	33,475,300 (75.31%)
200RE−_1	22,602,966	94.24%	45,205,932	34,189,903

				(75.63%)
200RE−_2	21,652,720	94.66%	43,305,440	33,097,162 (76.43%)
200RE−_3	22,119,123	94.25%	44,238,246	33,588,583 (75.93%)

Supplement Table S2. Concentrations with mean ± SE, fold changes, *p*-value for significantly changed metabolites identified between E+ and E− plants under 0 and 200 mM NaCl treatment.

**Metabolite names**	**Relative concentration under 0 mM NaCl treatment**		**Fold Changes**	***p*-value**
	**RE+**	**RE−**	**Log$_2$(0RE+/0RE−)**	
Lysine	0.129853±0.011318	0.024867±0.003721	2.38	0.004869
Salicin	0.3731±0.042647	0.027922±0.007228	3.74	0.008605
Noradrenaline	0.001863±0.000212	0.000276±0.000052	2.76	0.014194
4-aminobutyric acid	3.310951±0.189899	1.461069±0.177237	1.18	0.015635
Acetol	0.015531±0.000675	0.005762±0.001272	1.43	0.019795
Aconitic acid	0.014051±0.000888	0.004556±0.001093	1.62	0.020399
	**Relative concentration under 200 mM NaCl treatment**		**Fold Changes**	
	**RE+**	**RE−**	**Log$_2$(200RE+/200RE−)**	
Phytol	0.034894±0.003594	0.007928±0.00112	2.14	0.015186
myo-Inositol	0.355494±0.03257	0.116332±0.010909	1.61	0.017471
Piceatannol	0.00854±0.000875	0.00276±0.000339	1.63	0.030683
Glucose-6-phosphate	0.005946±0.000273	0.002725±0.000452	1.13	0.031895
Galactinol	0.004992±0.000867	$1.3e^{-08}$±$8.94e^{-10}$	18.55	0.040667
	**Relative concentration in roots of E+ plants**		**Fold Changes**	
	**200 mM NaCl treatment**	**0 mM NaCl treatment**	**Log$_2$(200RE+/0RE+)**	
Acetol	0.000179±$4.71e^{-05}$	0.015531±0.000675	-6.44	0.000003
Creatine	$2.11e^{-08}$±$2.59e^{-09}$	0.00054±0.00003	-14.64	0.000023

2-Aminoethanethiol	$2.11e^{-08}$±$2.59e^{-09}$	0.068416±0.004619	-21.63	0.000124
Aconitic acid	0.000976±0.000231	0.014051±0.000888	-3.85	0.000169
3,7,12-Trihydroxycoprostane	$2.11e^{-08}$±$2.59e^{-09}$	0.00351±0.000257	-17.34	0.000234
Oleic acid	$2.11e^{-08}$±$2.59e^{-09}$	0.420317±0.033439	-24.25	0.000443
D-Glyceric acid	0.012382±0.002852	0.586753±0.055516	-5.57	0.001777
Dehydroascorbic acid	$2.11e^{-08}$±$2.59e^{-09}$	0.016171±0.001607	-19.55	0.002116
L-Gulonolactone	0.041999±0.017145	0.573466±0.051886	-3.77	0.002641
Tetrahydrocorticosterone	$2.11e^{-08}$±$2.59e^{-09}$	0.005606±0.000621	-18.02	0.004225
Ascorbate	$2.11e^{-08}$±$2.59e^{-09}$	0.008485±0.000944	-18.62	0.00432
Resveratrol	$2.11e^{-08}$±$2.59e^{-09}$	0.000487±0.000055	-14.49	0.004916
Noradrenaline	$2.11e^{-08}$±$2.59e^{-09}$	0.001863±0.000212	-16.43	0.005001
Salicin	0.002076±0.000847	0.3731±0.042647	-7.49	0.005259
alpha-Santonin	0.000305±0.000124	0.008215±0.000916	-4.75	0.005781
Chlorogenic acid	0.000014±$3.06e^{-06}$	0.291122±0.035095	-14.31	0.006928
21-Hydroxypregnenolone	0.000633±$9.14e^{-05}$	0.142092±0.017636	-7.81	0.008366
Naringenin	$2.11e^{-08}$±$2.59e^{-09}$	0.002124±0.000269	-16.62	0.009021
2-Monopalmitin	0.004493±0.000586	$7.71e^{-09}$±$2.43e^{-10}$	19.15	0.010786
Orotic acid	0.000016±$6.63e^{-06}$	0.000784±0.000104	-5.59	0.013075
N-acetyl-5-hydroxytryptamine	0.000007±$1.92e^{-06}$	0.001974±0.000267	-8.05	0.013274
N-formyl-L-methionine	$2.11e^{-08}$±$2.59e^{-09}$	0.001159±0.000163	-15.74	0.015617
Citramalic acid	0.000008±$3.16e^{-06}$	0.005643±0.000846	-9.51	0.021577
N-acetyltryptophan	$2.11e^{-08}$±$2.59e^{-09}$	0.001637±0.000251	-16.24	0.023979
Leucine	0.62433±0.058184	0.180196±0.042858	1.79	0.030963
Cytidine	$2.11e^{-08}$±$2.59e^{-09}$	0.000784±0.000129	-15.18	0.032947
Butyraldehyde	$2.11e^{-08}$±$2.59e^{-09}$	0.002167±0.000373	-16.65	0.039139

	**Relative concentration in roots of E− plants**		**Fold Changes**
	**200 mM**	**0 mM**	**Log_2(200RE−/0RE−)**

	NaCl treatment	NaCl treatment		
3,7,12-Trihydroxycoprostane	$1.3e^{-08}\pm2.17e^{-09}$	0.003893±0.000649	-18.19	$1.59e^{-06}$
6-Phosphogluconic acid	0.000564±0.000094	0.002626±0.000438	-2.22	$8.41e^{-06}$
Raffinose	0.015498±0.002583	0.196301±0.032717	-3.66	$1.06e^{-05}$
Mucic acid	0.000609±0.000101	0.008638±0.00144	-3.83	$1.07e^{-05}$
D-(glycerol 1-phosphate)	0.005559±0.000926	0.075165±0.012528	-3.76	$1.40e^{-05}$
Maltotriose	0.000581±0.000097	0.046896±0.007816	-6.34	$1.48e^{-05}$
21-Hydroxypregnenolone	0.001093±0.000182	0.113922±0.018987	-6.7	$2.20e^{-05}$
Melezitose	0.001667±0.000278	0.012948±0.002158	-2.96	$2.81e^{-05}$
Cytidine	$1.3e^{-08}\pm2.17e^{-09}$	0.00118±0.000197	-16.47	$4.89e^{-05}$
Chlorogenic acid	$5.78e^{-06}\pm9.64e^{-07}$	0.313605±0.052267	-15.73	$7.87e^{-05}$
Oleic acid	$1.3e^{-08}\pm2.17e^{-09}$	0.73529±0.122548	-25.75	0.000271
Piceatannol	0.00276±0.00046	0.057824±0.009637	-4.39	0.000552
N-acetyl-L-leucine	$1.3e^{-08}\pm2.17e^{-09}$	0.000974±0.000162	-16.19	0.000571
D-Glyceric acid	0.013995±0.002332	0.282373±0.047062	-4.33	0.000863
Trehalose	0.252023±0.042004	0.014102±0.00235	4.16	0.000878
4-Hydroxybenzyl cyanide	$1.3e^{-08}\pm2.17e^{-09}$	0.001615±0.000269	-16.92	0.001012
Tetrahydrocorticosterone	$1.3e^{-08}\pm2.17e^{-09}$	0.004153±0.000692	-18.28	0.001103
2-aminoethanethiol	$1.3e^{-08}\pm2.17e^{-09}$	0.056668±0.009445	-22.05	0.001292
Naringenin	$1.3e^{-08}\pm2.17e^{-09}$	0.001932±0.000322	-17.18	0.002543
5-Aminoimidazole-4-carboxamide	0.000154±0.000026	0.000564±0.000094	-1.87	0.006041
5-Methoxytryptamine	0.001899±0.000316	0.009796±0.001633	-2.37	0.007805
Methylmalonic acid	0.030638±0.005106	0.004567±0.000761	2.75	0.01791
Tryptophan	0.105644±0.017607	0.006145±0.001024	4.1	0.018572
Leucine	0.324295±0.054049	0.058134±0.009689	2.48	0.018818
L-Gulonolactone	$1.3e^{-08}\pm2.17e^{-09}$	0.379241±0.063207	-24.79	0.0192
Creatine	0.000082±0.000014	0.000659±0.00011	-3.01	0.020066

Pipecolinic acid	0.019699±0.003283	0.002365±0.000394	3.06	0.020489
N-Acetyltryptophan	$1.3e^{-08}$±$2.17e^{-09}$	0.001089±0.000181	-16.35	0.02269
Orotic acid	0.000007±$1.11e^{-06}$	0.001216±0.000203	-7.51	0.02624
Guanosine	0.000695±0.000116	$5.52e^{-09}$±$2.17e^{-09}$	16.94	0.026719
Ascorbate	$1.3e^{-08}$±$2.17e^{-09}$	0.006532±0.001089	-18.94	0.027557
Glycolic acid	0.0486±0.0081	0.010371±0.001729	2.23	0.034815
Resveratrol	$1.3e^{-08}$±$2.17e^{-09}$	0.000347±0.000058	-14.7	0.049734
